# A review of remote sensing for potato traits characterization in precision agriculture

**DOI:** 10.3389/fpls.2022.871859

**Published:** 2022-07-18

**Authors:** Chen Sun, Jing Zhou, Yuchi Ma, Yijia Xu, Bin Pan, Zhou Zhang

**Affiliations:** ^1^Biological Systems Engineering, University of Wisconsin-Madison, Madison, WI, United States; ^2^Key Laboratory of Spectral Imaging Technology, Xi’an Institute of Optics and Precision Mechanics, Xi’an, China; ^3^School of Statistics and Data Science, Nankai University, Tianjin, China

**Keywords:** precision agriculture, remote sensing, satellite imagery, potato, unmanned aerial system, sensors

## Abstract

Potato is one of the most significant food crops globally due to its essential role in the human diet. The growing demand for potato, coupled with severe environmental losses caused by extensive farming activities, implies the need for better crop protection and management practices. Precision agriculture is being well recognized as the solution as it deals with the management of spatial and temporal variability to improve agricultural returns and reduce environmental impact. As the initial step in precision agriculture, the traditional methods of crop and field characterization require a large input in labor, time, and cost. Recent developments in remote sensing technologies have facilitated the process of monitoring crops and quantifying field variations. Successful applications have been witnessed in the area of precision potato farming. Thus, this review reports the current knowledge on the applications of remote sensing technologies in precision potato trait characterization. We reviewed the commonly used imaging sensors and remote sensing platforms with the comparisons of their strengths and limitations and summarized the main applications of the remote sensing technologies in potato. As a result, this review could update potato agronomists and farmers with the latest approaches and research outcomes, as well as provide a selective list for those who have the intentions to apply remote sensing technologies to characterize potato traits for precision agriculture.

## Introduction

Potato (*Solanum tuberosum* L.) is one of the most essential and commonly consumed non-grain food in the world. Due to its importance in the food industry supply chain, potato has become a dominant crop in many countries (Ortiz and Mares, 2017). For example, more than 23 million tons of potato tubers were harvested from about 940 thousand acres in the United States in 2019 (U. S. Department of Agriculture, 2020). Also, recently, the planting area and the total yield of potatoes have increased more than those of any other food crop in Africa. However, the continuously enhanced use of agricultural inputs (e.g., fertilizer and pesticide) could lead an environmental degradation, including the depletion of natural resources, polluted surface water and underground flows, and eutrophication (Kleinman et al., 2011; Konikow, 2015; Sishodia et al., 2017). Moreover, the over and/or inefficient use of water, fertilizers, pesticides, and herbicides for potato crops over the growing season causes economic losses and negative impacts on the environment (Hendricks et al., 2019). To constantly and sustainably meet the increasing needs for potato production, reducing environmental losses with improved potato productivity is the key topic in precision agricultural activities for potatoes (Delgado et al., 2019).

Traditionally, the effects of precision field activities, such as irrigation timing and frequency, fertilizer applications, planting dates, and population density, were determined through field surveying and/or biophysical modeling on crop growth and yield under different environmental conditions (Molahlehi et al., 2013; Al-Gaadi et al., 2016). Such methods commonly require visual examinations and destructive sampling and thus can be time-consuming with poor scalability. In the past few decades, the development of technologies, such as remote sensing, global positioning system, geographic information systems, artificial intelligence, has significantly promoted the applications in precision agriculture for enhancing crop production and quality while reducing the negative impacts on the environment in a more efficient way (Mulla, 2013). Precision agriculture has been gradually applied to monitor the crop growth status, generate auto-irrigation systems and disease detection systems in both developed and developing countries. Based on the interaction of electromagnetic radiation with soil and canopy reflectance, the remote sensing technique is considered as an effective tool in precision management for optimizing the decision-making process, which helps to improve profitable tuber yield and quality and minimize the negative impacts on the natural resources (Banerjee et al., 2020; Sanchez et al., 2020; Sun et al., 2020). Also, the data processing approaches, such as machine learning and image processing, have been widely used for extracting useful information from remotely sensed data (Dutta Gupta and Pattanayak, 2017; Oppenheim and Shani, 2017; Suh et al., 2018). Besides, cloud computing systems have been successfully used in data storing and processing for precision agriculture (Zhou et al., 2016b). Remote sensing systems, integrating advances in imaging, data processing, and computing technologies, thus have the potential to monitor the crop growth status and help make the decisions for crop management (Pavón-Pulido et al., 2017; Say et al., 2017).

In recent years, information-based precision agriculture has been successfully applied to explore potato crop traits and field phenotyping (Fortin et al., 2011; Sugiura et al., 2016; Franceschini et al., 2017a; Pei et al., 2019). Potatoes are stem tubers that are concealed in soils over the growing season. After being seeded in the soil, the top sides of the tuber produce shoot that grow into typical stems and leaves, and the undersides produce roots (Khayatnezhad et al., 2011). By establishing the relationship between traits of the aboveground parts and the underground tubers, remote sensing techniques have great potential in estimating the tuber growth conditions and predicting tuber yield prior to harvest (Shayanowako et al., 2014). Compared with traditional sampling methods, remote sensing is capable of providing a non-destructive and efficient way to collect the multi-temporal reflectance data of the aboveground plants for monitoring the biomarkers of potato crops (Nikolakopoulos et al., 2017; Feng et al., 2020). Past studies have focused on the specific applications of potatoes in precision agriculture using remote sensing approaches, such as tuber yield prediction (Akhand et al., 2016; Zaeen et al., 2020), above-ground biomass (AGB) estimation (Li B. et al., 2020; ten Harkel et al., 2020), water stress detection (Rud et al., 2014), nitrogen stress assessment (Bohman et al., 2019), and disease detection (Nebiker et al., 2016; Franceschini et al., 2017b,^2019^; Polder et al., 2019). Most of these studies demonstrated the strengths of remote sensing techniques along with their limitations and future challenges for the applications in potato production.

The primary purpose of this review is to provide a comprehensive background and knowledge on applications of remote sensing technologies in potato trait characterization in the context of precision agriculture. Both Web of Science and Google Scholar databases were used for literature review with keywords or topics of potato remote sensing from 2010 to the present. This paper is organized in the following main aspects: (1) compare the strengths and limitations of a variety of imaging sensors and remote sensing platforms and (2) summarize the remote sensing techniques adopted for the specific applications of potatoes in precision agriculture.

## Imaging sensors

Remote sensing for crop trait characterization broadly involves a series of techniques in sensing, recording, analyzing, and/or modeling. When incident radiation (light source or natural light) hits plant surface, the radiation will either be absorbed, transmitted, or reflected. The reflected radiation is expressed as the electromagnetic spectrum with a range of wavelengths. The sensing technologies include developing devices to capture the reflected spectrum and convert them into readable signals for further analysis (Xie and Yang, 2020).

When choosing sensors for a specific purpose, image temporal, spatial, and spectral resolutions are the three main factors to consider. Temporal resolution refers to the time window between two images over the same place. Spatial resolution refers to the size of the smallest objects that can be captured and displayed in the imagery. Spectral resolution refers to the ability of a sensor to measure specific wavelengths of the electromagnetic spectrum and it is mainly decided by the sensor types.

Within the context of potato trait characterization, a variety of sensors has been developed and applied for different purposes. The sensors, varying in their spatial and temporal resolutions, mainly include visible Red-Green-Blue (RGB) cameras, multi- and hyper-spectral cameras, thermal infrared (TIR) cameras, and light detection and ranging (LiDAR) sensors. In this section, we summarized the sensors that have been applied in characterizing potato traits with comparisons of their strengths and limitations.

### Visible red-green-blue cameras

Visible RGB cameras receive lights in red (564–580 nm), green (534–545 nm), and blue (420–440 nm) board spectral bands in the visible spectrum regions to emulate human vision. Due to the broad applicability of RGB cameras in all the fields, they are well-developed products by private sectors with a large selective range in price, sensor sizes, lens availability, data storage, and transmission. For characterizing crop traits, the RGB cameras generally have the best performance in providing high spatial-resolution images with the ability to be associated with various sensing environments, which makes them the most commonly adopted sensors in the areas of remote sensing.

Applications in potato trait characterization have been leveraging the advantages of RGB images in different ways. In potato, studies have been conducted on quantifying disease severity using the RGB images by detecting pixels of diseased leaves with that of healthy leaves and soil. It was shown that the diseased leaves could be clearly distinguished from the transformed color spaces (Sugiura et al., 2016) or spectral indices (Gibson-Poole et al., 2017; Siebring et al., 2019) from the RGB color space. Also due to the contrast between plant and soil background captured by the RGB image, promising applications have been seen in detecting potato emergence (Gibson-Poole et al., 2018; Li et al., 2019) and calculating ground vegetation coverage (Gibson-Poole et al., 2018). Other than the color-based features, high resolution images from the RGB cameras can be used to reconstruct three-dimensional (3D) dense point models using photogrammetry methods. Plant height is then obtained by subtracting the digital elevation model (DEM) which represents the absolute elevation of the bare ground from the digital surface model (DSM) which represents the absolute height of the plant canopies. Li B. et al. (2020) compared the performance in estimating potato plant height from the RGB 3D models with the plant height regressed from the hyperspectral reflectance and the results showed that the RGB 3D models lead to a higher correlation with the ground truth data [Coefficient of determination *R*^2^ = 0.93, root mean square error (RMSE) = 6.39 cm] than the full spectra regression (*R*^2^ = 0.85, RMSE = 7.24 cm).

Compared with other sensors, the RGB cameras provide an intuitive way to visualize and quantify crop traits. RGB cameras are typically low-cost, lightweight, and can be easily mounted on various platforms for data collection. However, three spectral bands and the broad bandwidth of the RGB cameras limit their ability to receive crop traits other than human perceptions and consequently their applications in sensing more complicated crop traits, such as yield and nutrient deficit. Besides, it requires extra efforts to remove background noises from the RGB images under certain situations when the plants have similar visual colors to the background or when plants are covered with shadows and other plants (Li et al., 2014).

### Multi- and hyper-spectral cameras

Multi- and hyper-spectral cameras provide information with higher spectral resolutions than the RGB cameras and spectral bands additional to the visible range. The multispectral cameras usually consist of a small number (less than 10) of discrete wavebands in the visible-near infrared range from 400 to 1,000 nm. The spectral wavebands usually are narrower RGB bands and those with prior knowledge of their sensitivity in receiving important crop information (Hunt et al., 2013), such as red edge (700–730 nm) and NIR (760–900 nm). There are two types of multispectral cameras currently used in precision agriculture, that is, customized and commercial off-the-shelf cameras. As suggested by the name, the customized camera has the internal NIR filter removed from a visible RGB camera and is then replaced with an external filter to alter the camera spectral sensitivity (Zhou et al., 2016a), for example, from R-G-B to R-G-NIR. The commercial multispectral cameras are explicitly made with an interference filter to block or transmit specific lights (Xie and Yang, 2020). Compared with the customized camera, the commercial ones can provide more than three wavebands with other selectable spectral regions, but with lower spatial resolutions (Table 1).

**TABLE 1:** Sensors and their applications in potato trait characterization.

Sensors	Manufacturers	Models	Image resolution	Spectral range (nm)	No. of bands	Applications
Visible red–green–blue (RGB)	DJI	Phantom 4 Pro	5,472 × 3,648	400–700	3	Plant height estimation (Li B. et al., 2020); Disease detection (Sugiura et al., 2018); Emergence estimation (Li et al., 2019)
	Sony	NEX-5N	1,920 × 1,080			Disease detection (Sugiura et al., 2016)
	Canon	A2200	4,320 × 3,240			Disease detection (Gibson-Poole et al., 2017)
		ELPH 115 IS	4,608 × 3,456			Growth status prediction (Gibson-Poole et al., 2018)
	Panasonic	GX1	4,592 × 3,448			Disease detection (Siebring et al., 2019)
Multispectral	Tetracam	Micro-MCA RGB + 3	1,280 × 1,024	350–950	6	Yield prediction (Tanabe et al., 2019)
		ADC Lite	2,048 × 1,536	520–920	3	Crop scouting (Théau et al., 2020); Insect detection (Hunt et al., 2016); nitrogen stress estimation (Hunt et al., 2016, 2018; Hunt and Rondon, 2017)
		Mini-MCA	1,280 × 1,024	450–1,000	6	Beetle damage assessment (Hunt and Rondon, 2017)
	Kodak	Megaplus 4.2i cameras (customized)	2,024 × 2,044	400–1,000	3	Yield prediction (Sankaran et al., 2015)
	Canon	S95 (customized)	3,648 × 2,736	400–1,000	4	Chlorophyll content estimation (Elarab et al., 2015)
		S110 (customized)	4,000 × 3,000	400–1,000	3	Disease detection (Nebiker et al., 2016; Duarte-Carvajalino et al., 2018)
		Powershot ELPH 340 HS (customized)	4,608 × 3,456	400–1,000	3	Growth status estimation (Théau et al., 2020)
		ELPH 110 (customized)	4,608 × 3,456	400–1,000	3	Hail damage assessment (Zhou et al., 2016a)
	Redlake	MS4100	1,920 × 1,080	400–1,000	3	Nitrogen stress prediction (Nigon et al., 2014)
	MicaSense	RedEdge	1,280 × 960	400–1,000	5	Structural change (Angulo-Morales et al., 2020); Radiation use efficiency assessment (Peng et al., 2021)
Hyperspectral	Headwall	Nano-Hyperspec	640 × 1	400–1,000	270	Yield prediction (Li B. et al., 2020; Sun et al., 2020)
	Specim	ImSpector V10 2/3”	Depending on the combined imager	400–950	61	Chlorophyll content assessment, leaf area index (LAI) estimation and ground cover assessment (Franceschini et al., 2017a); nitrogen stress detection (Nigon et al., 2015); canopy changes assessment (Franceschini et al., 2017b); crop growth assessment (Gevaert et al., 2014); Reflectance anisotropy measurements (Roosjen et al., 2016)
	Cubert	UHD 185	1,000 × 1,000	450–950	125	Chlorophyll content assessment (Li C. et al., 2020)
	Rikola	Fabry-Perot interferometer	1,010 × 1,010	500–900	16	Disease detection (Franceschini et al., 2019); Chlorophyll content assessment and LAI estimation (Roosjen et al., 2018); Reflectance anisotropy assessment (Roosjen et al., 2017)
Thermal infrared	Infrared cameras	Microbolometer	640 × 480	7,000–14,000		Chlorophyll content assessment (Elarab et al., 2015)
	Wilsonville	TAU 640	640 × 512	7,500–13,500		Crop scouting (Théau et al., 2020)
	FLIR	E60	320 × 240	7,500–13,000		Water status (Cucho-Padin et al., 2020)
	FLIR	SC2000	320 × 240	7,500–13,000		Water status (Rud et al., 2014)
	FLIR	SC655	640 × 480	7,500–13,000		Water status (Rud et al., 2014)
	JeanOptics	IDM200	640 × 480	7,500–13,000		Water status (Rud et al., 2014)
	Fluke	Ti-32	320 × 240	7,500–14,000		Water status (Elsayed et al., 2021a)
	Fluke	TiR1	160 × 120	7,500–14,000		Water status (Gerhards et al., 2016)
	Telops	HyperCamLW	320 × 256	7,700–11,500		Water status (Gerhards et al., 2016)
	Palmer Wahl Instruments	HSI3000	160 × 120	8,000–14,000		Water status (Ghazouani et al., 2017)
Light detection and ranging (LiDAR)	RIEGL	VUX-1				Crop height and biomass estimation (ten Harkel et al., 2020)

By integrating both spectroscopic and imaging techniques into one system, hyperspectral cameras provide 3D hyperspectral cubes that contain both contiguous spectral information and two-dimensional spatial information. There are four types of hyperspectral cameras based on their image acquisition methods, namely point scanning, line scanning, area scanning, and snapshot cameras (Wu and Sun, 2013). The line scanning, also known as the pushbroom cameras, records a line of an image at each time and then forms the whole image by scanning along the other direction. The line scanning cameras are particularly suitable for being carried on imaging platforms that follow certain moving patterns (e.g., zigzag paths) to cover a large field and thus are the most popular ones in precision agricultural applications (Xie and Yang, 2020). Compared with the multispectral camera, hyperspectral cameras provide a real spectrum in every image pixel, allowing to calculate advanced vegetation indices (VIs) and estimate unperceived crop phenotypes. However, hyperspectral cameras have more restrictions on reflectance calibration procedures and moving patterns and they are more costly both in the devices and data storage (Li et al., 2014).

The spectral cameras have been widely used to predict potato leave biomass (Li B. et al., 2020; Peng et al., 2021)/tube yield (Sankaran et al., 2015; Tanabe et al., 2019; Li B. et al., 2020; Sun et al., 2020), detect canopy damage/disease (Nebiker et al., 2016; Hunt and Rondon, 2017; Duarte-Carvajalino et al., 2018; Franceschini et al., 2019), and estimate potato biophysical properties, such as chlorophyll content (Elarab et al., 2015; Franceschini et al., 2017a; Li C. et al., 2020), nitrogen concentrations (Nigon et al., 2014, 2015; Hunt and Rondon, 2017; Hunt et al., 2018), and leaf area index (LAI, Franceschini et al., 2017a). Among them, disease detection is the common application of visible RGB and spectral cameras. Unlike separating the healthy and unhealthy leaf areas in RGB images, spectral cameras are used to investigate the relationship between spectral measurements and disease severity at plot/field level. For example, the normalized difference vegetation index (NDVI) combined with spectral reflectance values in NIR and R was able to highlight sites infested by blight disease in a large potato field (Nebiker et al., 2016). Atherton et al. (2015) also reported that heavily diseased plants had significantly different spectral profiles from healthy plants.

### Thermal infrared cameras

Radiation emitted by objects in the longwave infrared range (8–1.3 μm) is proportional to their surface temperature, which can be exploited for remote measurements of plant canopy temperature using TIR cameras (Kuenzer and Dech, 2013; Williams et al., 2018). Plant canopy temperature has been used as an indicator of plant stomatal traits (e.g., stomatal conductance, stomatal aperture, or leaf porosity) because leaves surfaces are cooled by evaporation, and stomatal opening or higher transpiration rates lead to high evaporation rates (i.e., decreased temperatures; Deery et al., 2019). In plant science and agronomy, measuring plant leaf temperature using thermal imaging has long been used to study the mechanisms behind plant water relations (Chaerle and Van Der Straeten, 2000), plant physiology processes (Jones et al., 2002; Aldea et al., 2006a; Grant et al., 2010), plant responses to pathogens (Chaerle et al., 1999) or environmental stressors (Jones et al., 2009), and plant photosynthetic rate (Aldea et al., 2006b; Vítek et al., 2020). In the area of precision agriculture, substantial interests have been witnessed in irrigation scheduling (Li et al., 2022), yield prediction (Bellis et al., 2022), evapotranspiration estimation (Lu et al., 2022; Wolff et al., 2022), pre-symptomatic diagnosis (Cohen et al., 2022), and stress tolerance (Zhou et al., 2020; Degani et al., 2022; Rippa et al., 2022).

In potato applications, TIR cameras are primarily used to evaluate potato water status. Plant water stress indicators, for example the Crop water stress index (CWSI), were calculated using TIR image-derived temperature and validated by comparing with biophysical measurements of plant water status. Related studies showed that the TIR image-based indices highly agreed with the ground measurements and were able to reflect the effects of different irrigation treatments (Rud et al., 2014; Gerhards et al., 2016; Cucho-Padin et al., 2020; Elsayed et al., 2021a). In some other applications, the TIR cameras were used as an aid tool for spectral cameras for detecting pest stress (Théau et al., 2020) or assessing chlorophyll content (Elarab et al., 2015).

The TIR cameras are the most effective tool in remotely sensing plant leaf/canopy temperature and water-related status. In practical, pre-calibration and post-correction procedures are required for receiving reliable temperature readings from the TIR cameras especially under changing ambient environmental factors such as air temperature, humidity, and wind speed (Jones et al., 2009; Jin et al., 2021). Additionally, the low spatial resolutions of the TIR cameras (Table 1) make them easily affected by image background temperature. It is also reported by Théau et al. (2020) that spectral indices (including visible ones) would be preferred over the TIR indices when both performed similarly in detecting various plant characteristics.

### Light detection and ranging system

Light detection and ranging (or laser scanner) sensors are used to obtain depth information of targeted objects. A LiDAR device has a single-beam laser (narrowband in 600–1,000 nm) that emits a light pulse and a receiver measures the propagation time of the light pulse from emission to reflection. The time is then converted into a distance given the known speed of light (ten Harkel et al., 2020). LiDAR devices are lightweight with compact sizes and thus can be easily integrated on different platforms. Unmanned aerial vehicle (UAV)-based LiDAR systems have been applied to map the 3D structure of the plant canopy and topography of the landscape (Lin et al., 2019; ten Harkel et al., 2020). UAV-based LiDAR devices were reported to deliver accurate estimations of plant height with the RMSE of 0.02, 0.07, and 0.12 m compared with the manual measurements on snap bean crops (Zhang et al., 2021), sugar beet, and potato (ten Harkel et al., 2020), respectively. However, LiDAR devices can be expensive and require longer imaging times, compared to other stereo vision methods from images for 3D mapping.

## Remote sensing platforms for data collection

In precision agriculture, a variety of remote sensing platforms have been developed and applied for different purposes, mainly including ground and aerial platforms. The ground platforms provide higher spatial and temporal resolution imagery than the aerial platforms and generally do not require a specific certificate to operate. Depending on the structural differences, four types of ground platforms are often seen, namely pole-based, cable-suspended, tractor, and gantry platforms (Li et al., 2021b). However, due to the limited spatial coverage, vibrations, and causes of soil compaction, ground platforms are challenged to be used in large-scale applications (Sankaran et al., 2015), such as potato precision management. Thus in this section, we focused on the aerial platforms that have been largely applied in precision potato management, that is, satellite-based and UAV-based platforms, with comparisons of their strengths and limitations.

### Satellite-based remote sensing platforms

Satellite imagery, as one of the remote sensing means, began more than half a century ago and has been used efficiently in multiple agricultural applications (Ma et al., 2021; Wang Y. et al., 2021). Its major benefits include non-destructive capture of the earth surface at wide geographical regions (hundreds of square kilometers in a single image), selectable revisit time (1 day–2 weeks), and sensors with high spatial and spectral resolutions (Zhang et al., 2020). In recent years, a number of studies have investigated the potential of using satellite-based remote sensing platforms for precision potato management activities. The specifications for eleven representative platforms and their corresponding applications in precision potato management have been summarized in Table 2.

**TABLE 2:** Satellite-based remote sensing platforms and their applications in potato trait characterization.

Satellite	Instrument	Spatial resolution (m/pixel)	Temporal resolution (days)	Spectral range (nm)	No. of bands	Applications
Terra	Moderate Resolution Imaging Spectroradiometer (MODIS)	250–1,000	1–2	400–1,440	36	Yield prediction (Salvador et al., 2020); disease assessment (Dutta et al., 2014)
	Advanced Spaceborne Thermal Emission and Reflection Radiometer (ASTER)	15–90	4–16	500–12,000	14	Herbicide rate assessment (Van Evert et al., 2012)
Planet scope	PS2	3	1	455–860	4	Yield prediction (Abou Ali et al., 2020)
Resourcesat-1	Advanced Wide Field Sensor (AWiFS)	56	5	520–1,700	3	Disease assessment (Dutta et al., 2014)
Sentinel-2	Multispectral Instrument (MSI)	10–60	5	400–2,190	13	Leaf area index (LAI) and chlorophyll content estimation (Herrmann et al., 2011; Clevers and Kooistra, 2012; Clevers and Gitelson, 2013; Clevers et al., 2017); Radiation utilization assessment (Peng et al., 2021); Yield prediction (Al-Gaadi et al., 2016; Gómez et al., 2019; Abou Ali et al., 2020); crop mapping (Ashourloo et al., 2020)
Sentinel-3	The Ocean and Land Color Instrument (OLCI)	300	27	400–1,020	21	Nitrogen stress prediction (Clevers and Gitelson, 2013)
Landsat-5	Thematic Mapper (TM)	30–120	16	450–12,500	7	Yield prediction (Newton et al., 2018); LAI and Crop height estimation (Papadavid et al., 2011)
Landsat-7	Enhanced Thematic Mapper Plus (ETM+)	15–60	16	450–12,500	8	Yield prediction (Newton et al., 2018; Awad, 2019); LAI and Crop height estimation (Papadavid et al., 2011)
Landsat-8	Operational Land Imager (OLI)	30	16	433–1,390	9	Yield prediction (Al-Gaadi et al., 2016; Newton et al., 2018; Awad, 2019)
Formosat-2	Remote Sensing Instrument	8	1	450–900	4	Spectral-Temporal Response Surfaces (STRS) estimation (Gevaert et al., 2015); Crop growth assessment (Gevaert et al., 2014)
Proba-1	Compact High Resolution Imaging Spectrometer (CHRIS)	18	2	400–1,050	19	LAI estimation (Atzberger and Richter, 2012; Delegido et al., 2013)
Polar Orbiting Environmental Satellites (POES)	The Advanced Very High-Resolution Radiometer (AVHRR)	1,100	7/14	580–12,500	5	Yield prediction (Khan et al., 2012; Akhand et al., 2016)

Among all the selected satellite missions, the Landsat program involves a series of satellites that have been successfully used in agricultural monitoring because of the availability of historical data of the Earth’s surface for over 40 years. The most recent Landsat-8 was launched in 2013 with two imagers (Operational Land Imager and Thermal Infrared Sensor) that provide spectral observations in nine spectral bands at a 30-m spatial resolution. Several studies thus adapted the high-resolution observations from Landsat-8 for potato yield prediction (Al-Gaadi et al., 2016; Newton et al., 2018; Awad, 2019).

Besides the Landsat missions, Sentinel-2 is another popular platform for agriculture monitoring. For the potato management, Sentinel-2 imagery has been used for LAI and chlorophyll content estimation (Herrmann et al., 2011; Clevers and Kooistra, 2012; Clevers and Gitelson, 2013; Clevers et al., 2017), radiation utilization assessment (Peng et al., 2021), yield prediction (Al-Gaadi et al., 2016; Gómez et al., 2019; Abou Ali et al., 2020), and crop mapping (Ashourloo et al., 2020). Launched in 2015 by Copernicus Programme, Sentinel-2 systematically acquires optical imagery with global coverage. It is equipped with a push-broom multi-spectral instrument (MSI) which provides optical observations at 13 spectral bands ranging from visible (VIS) to short-wave infrared (SWIR) at high spatial resolutions (10–60 m/pixel). Like Landsat-8, Sentinel-2 also provides a global coverage but with a much higher temporal resolution (Table 1), which makes Sentinel-2 the most widely used satellite platform in potato precision management.

Other satellite platforms with unique specifications in one of the resolutions have been used to address specific purposes in potato management. For example, Terra Moderate Resolution Imaging Spectroradiometer has the highest spectral resolution of 36 bands in the range between 400 and 1,440 nm and thus the benefits for detecting the abnormal reflectance from leaves in the narrow bands for potato disease assessment (Dutta et al., 2014). Planet Scope provides images with the highest spatial resolution (3 m/pixel) every day through a constellation of about 130 low-orbit satellites. Though costly (∼$ 218 per 100 km^2^) compared to the publicly launched satellites, its imagery has been used as a good source for the decision-making at field-level and plot-level potato management on a daily basis (Abou Ali et al., 2020).

### Unmanned aerial vehicle-based remote sensing platforms

Over the decades, UAV-based platforms have been a popular approach in precision agriculture. UAVs refer to devices with flying capacity without humans onboard (Eisenbeiss, 2004; Sankaran et al., 2015). Other than the UAV and its accessories (e.g., batteries and remote controller), UAV-based remote sensing platforms for agricultural purposes generally include onboard sensors for collecting data, a gimbal for motion correction and stabilization, and a flight planning App installed in a smart mobile device for automated flight control. To deliver precisely geo-referenced data, UAV platforms are typically coupled with ground control points (GCPs) with known global navigation satellite system positions.

The UAV platforms are available in several types with distinguishing features. The most widely used UAVs in precision agriculture are multi-rotor copters or fixed-wings (Li et al., 2021b). Multi-rotor copters can be quadcopters, hexacopters, or octocopters depending on the number of propellers they have. Multi-rotor copters are driven by the pairs of propellers to create thrust downward and directed by changing the thrust of one or more propellers. Thus, they are able to hover, take off and land vertically and have no lower limits on flight altitude. Fixed-wing UAVs, like airplanes, have a pair of rigid wings and a thruster at back. The lift of fixed-wing UAVs comes from the air pressure differences between the upper and lower sides that are generated passively as its wings cut through the air at a specific angle when being pushed forward. Fixed-wing UAVs are fast and energy-efficient, and thus allow much larger ground coverage than the multi-rotor copters. On the other hand, they do not have the capability to hover and fly close to the ground. A list of popular UAV models and their applications in potato applications are summarized in Table 3.

**TABLE 3:** UAV-based remote sensing platforms and their applications in potato traits characterization.

UAV type	Manufacturer	Model	On board sensors	Max. speed (km/h)	Duration (min)	Applications
Quadcopters	DJI	Phantom 4 Pro	Visible RGB	50–72	30	Plant height estimation (Li B. et al., 2020); Disease detection (Sugiura et al., 2018); Emergence estimation (Li et al., 2019)
Quadcopters	DJI	Inspire 2	RGB; Multispectral	94	25	Yield prediction (Li et al., 2021a)
Quadcopters	3D Robotics	Solo	Visible RGB	80	25	Growth status prediction (Gibson-Poole et al., 2018)
Quadcopters	DJI	Matrice 100 *	Multispectral	61–79	20–40	Radiation use efficiency assessment (Peng et al., 2021)
Hexacopter	DJI	Matrice 600 Pro	Customizable	65	25	Yield prediction (Sun et al., 2020)
Hexacopter	Tarot	680 Pro	Customizable	–	–	Structural change (Angulo-Morales et al., 2020)
Octocopter	Aerialtronics	Altura AT8 *	Customizable	–	–	Chlorophyll content assessment, leaf area index (LAI) estimation and ground cover assessment (Franceschini et al., 2017a); Crop growth assessment (Gevaert et al., 2014); Chlorophyll content assessment and LAI estimation (Roosjen et al., 2018); Reflectance anisotropy measurements (Roosjen et al., 2016, 2017); Disease detection (Siebring et al., 2019)
Octocopter	Riegl	RiCOPTER	Visible RGB; LiDAR	30	30	Crop height and biomass estimation (ten Harkel et al., 2020)
	GmbH HiSystems	Unspecified	Visible RGB	–	–	Disease detection (Sugiura et al., 2016, 2018)
Fixed-wing	Trimble	UX5 HP	Visible RGB	80	35	Plant height estimation (De Jesus Colwell et al., 2021)
Fixed-wing	senseFly	eBee micro *	Visible RGB; Multispectral	110	25–50	Disease detection (Nebiker et al., 2016)

*Better versions are available now.

Selection of UAVs is conditional on specific tasks, including field area, spatial resolution, flexibility for carrying various cameras, and costs. Among all UAV types, quadcopters are the most popular as they are light weight and easy to operate. The DJI Phantom series with high-resolution motion RGB cameras have been used in applications for obtaining plant structural traits (Li B. et al., 2020) and recently they have an updated multispectral version for those who favor spectral measurements. Hexacopters generally are not sold with onboard sensors but offer interfaces to power and control third-party cameras. Thus the hexacopters are often used to carry multi- or hyper-spectral cameras for perceiving complicated crop traits such as yield responses (Feng et al., 2020; Zhou et al., 2021b) or stress (Zhou et al., 2020, 2021a). Similar to the hexacopter, octocopters are flexible in carrying multiple sensors and other devices for durable flight. The most widely accepted octocopter is the Altura AT8 (Aerialtronics, South Holland, Netherlands) which was used to carry a hyperspectral imaging system (2.0 kg in total) as the original version of the Hyperspectral Mapping System (HYMSY, Suomalainen et al., 2014). The HYMSY system costs around $12,769 and is largely used in potato studies conducted in European. On the opposite to multi-rotor UAVs, the fixed-wings offer a solution to the need of covering large fields because of their nature of high flight speed and altitude. Cameras on fixed-wings should have high frame rates and short exposure time to avoid blurred images caused by the high flight speed (Li et al., 2021b). As products evolve, fixed-wings vendors now offer a variety of versions with dual or triple cameras for agricultural purposes.

## Applications

Precision agricultural management is the key to reducing environmental impact and enhancing agricultural sustainability (Zhang et al., 2021). Within the context of potato management, a variety of activities are employed to ensure its productivity and quality throughout the growing cycle. Some of these activities heavily rely on *in situ* field monitoring of potato growing conditions, field environmental conditions and diseases, followed by in-season decision-making on irrigation, fertilization, pesticides and off-season guidance on storage, sell, and post-harvest field management. Remote sensing techniques have been explored and applied to directly measure crop yield, disease damages, and some key indicators of potato growth. In this section, we reviewed the applications of these techniques in potato management, including tuber yield prediction, AGB estimation, water deficit stress assessment, nitrogen concentration estimation, and disease detection. Some key traits that are closely related to potato growth were also summarized, namely plant height, LAI, chlorophyll, and emergence detection and plant counting. The details of the applications can be found in Table 4.

**TABLE 4:** Applications of remote sensing technologies in Potato traits characterization.

Application		Platform	Sensor	Model	Sensor-derived feature	References
**Tuber yield prediction**		UAV	Hyperspectral camera	OLS; Ridge; PLSR; SVR; RF; AdaBoost	Full spectra	Sun et al., 2020
			RGB, multi- and hyper- spectral cameras	RF; PLSR	13 narrow-band VIs	Li B. et al., 2020
			Multispectral camera	ANN	NDVI	Tanabe et al., 2019
				RF; SVR	15 wide-band VIs	Li et al., 2021a
		Satellite	Terra MODIS	LR	VIs	Bala and Islam, 2009
				RF; SVM; LR	NDVI	Salvador et al., 2020
			Sentinel-2	9 machine learning models	54 features including band reflectance and VIs	Gómez et al., 2019
			Landsat-8; Sentinel-2	LR	NDVI; SAVI	Al-Gaadi et al., 2016
**Above-ground biomass (AGB) estimation**		UAV	RGB and Hyperspectral camera	RF; PLSR	13 narrow-band VIs; Image-based plant height	Li B. et al., 2020
			LiDAR	3D Profile Index (Jimenez-Berni et al., 2018)	LiDAR point cloud	ten Harkel et al., 2020
		Handheld	Hyperspectral spectrometer	PLSR, MLR, RF	NDSI, RSI, DSI	Pei et al., 2019
				GA; ANFIS	20 narrow-band VIs	Elsayed et al., 2021b
				RF	12 narrow-band VIs	Yang et al., 2021
			TIR and RGB cameras	MLR; ANFIS	NRCT and 12 wide-based VIs	Elsayed et al., 2021a
		UAV; Satellite; handheld	Multispectral camera; Sentinel-2; multispectral spectrometer	Carnegie–Ames–Stanford approach	RVI and raw bands	Peng et al., 2021
**Water deficit stress assessment**		UAV; handheld	TIR and RGB cameras	LR	CWSI	Rud et al., 2014
		Handheld	TIR camera	–	CWSI	Cucho-Padin et al., 2020
				MLR; ANFIS	NRCT	Elsayed et al., 2021a
				LR	CWSI	Ghazouani et al., 2017
**Nitrogen concentration estimation**		UAV	Multispectral camera	LR	NDVI; GNDVI	Hunt and Rondon, 2017
		MAV	Multispectral camera	LR	GNDVI; GRVI; NDVI; NG	Nigon et al., 2014
			Hyperspectral spectrometer	PLSR	NG and 6 narrow-band VIs	Nigon et al., 2015
		Handheld	Multispectral radiometer	LR	8 wide-band VIs	Clevers and Gitelson, 2013
			Hyperspectral spectrometer	LR	4 SWIR-based indices	Herrmann et al., 2011
			Multispectral camera	LR	NDVI; RVI; RRE; RRE/GC	Zhou et al., 2018
**Disease detection**	PVY	Ground vehicle	RGB-Depth camera; hyperspectral line scan camera	FCN	Full spectra	Polder et al., 2019
		Handheld	Hyperspectral spectrometer	SVM	5 EM wavelength segments	Griffel et al., 2018
				PLS-DA	NDSI for all combinations of wavelengths	Couture et al., 2018
	Late blight	UAV	RGB camera	Color threshold	HSV color space	Sugiura et al., 2016
			Multispectral camera	–	NDVI	Nebiker et al., 2016
				CNN; SVR; RF	Differences between G and B as well as between NIR and G	Duarte-Carvajalino et al., 2018
		Satellite	Terra MODIS; AWiFS	Clustering	NDVI and LSWI	Dutta et al., 2014
		Handheld	Hyperspectral spectrometer	ANOVA; Stepwise Discriminant Analysis	NDVI; SR; SAVI; REI; and full spectra	Ray et al., 2011
	Early blight	Ground vehicle	Hyperspectral camera	PLS-DA; SVM	Full spectra; five wide-band VIs	Van De Vijver et al., 2020
		Handheld	Hyperspectral spectrometer	PCA; spectral change analysis; and PLSR	Full spectra	Atherton et al., 2015
**Plant height**		UAV	RGB camera and hyperspectral sensor	SfM; RF and PLSR	RGB images and 13 narrow-band VIs	Li B. et al., 2020
			LiDAR scanner	3DPI	3D-point cloud	ten Harkel et al., 2020
			RGB camera	SfM; “trace all triangles” method and “do not track breaklines” method	RGB images	De Jesus Colwell et al., 2021
**Leaf area index (LAI)**		Handheld	Hyperspectral spectrometer	PLSR	Full spectra NDVI; REIP	Herrmann et al., 2011
		Handheld; satellite	Hyperspectral spectrometer; Landsat TM/ETM+	Linear, exponential, and logarithmic regression	NDVI; SAVI; WDVI	Papadavid et al., 2011
		UAV	Multispectral camera	PROSAIL model inversion	Multi-angular multispectral data	Roosjen et al., 2018
		Satellite	Sentinel-2 MSI	LR	WDVI	Clevers et al., 2017
			Landsat-8 OLI; Sentinel-2 MSI	LR; SNAP model	SAVI; NDVI; EVI2; SeLI	Mourad et al., 2020
**Leaf chlorophyll content (LCC)**		Handheld	RGB camera	ANN; LR	Mean brightness parameters and mean brightness ratio	Dutta Gupta et al., 2013
			Hyperspectral spectrometer	PLSR	Full spectra; normalized spectra; MFs spectra	Liu et al., 2020
			Multispectral camera	LR; MLR	mean and std of R and G	Borhan et al., 2017
		Handheld; satellite	Hyperspectral spectrometer; REIS	LR	TCARI/OSAVI, TCI/OSAVI and CVI	Kooistra and Clevers, 2016
		UAV; satellite	Hyperspectral spectrometer; FORMOSAT-2	LR	Spectral–temporal response surfaces	Gevaert et al., 2015
**Emergence detection and plant counting**		UAV	RGB camera	Mask R-CNN	RGB images	Machefer et al., 2020
				RF	Morphological features	Li et al., 2019
			Multispectral camera	LR	NDVI	Sankaran et al., 2017

Abbreviations for Table 3: OLS, ordinary least squares; PLSR, partial least square regression; SVR, support vector regression; RF, random forest; VI, vegetation index; ANN, artificial neural network; NDVI, normalized difference vegetation index; LR, linear regression; SVM, support vector machine; SAVI, soil adjusted vegetation index; NDSI, normalized difference snow index; RSI, ratio spectral index; DSI, difference spectral index; MLR, multiple linear regression; GA, genetic algorithm; ANFIS, adaptive neuro-fuzzy inference system; NRCT, normalized relative canopy temperature; RVI, ratio vegetation index; CWSI, crop water status index; GNDVI, green normalized difference vegetation index; GRVI, green and red ratio vegetation index; NG, normalized green; RRE, ratio red edge vegetation index; GC, canopy cover; FCN, fully convolution neural network; EM, electromagnetic spectrum; PLS-DA, partial least-squares discriminate analysis; CNN, convolution neural network; G, green; B, blue; NIR, near infrared; LSWI, integration of leaf water content index; ANOVA, analysis of variance; SR, simple ratio; REI, red edge index; PCA, principal components analysis; SfM, structure from motion; 3DPI, 3D profile index; REIP, red-edge inflection point; WDVI, weighted difference vegetation index; PROSAIL, the coupling of the PROSPECT and SAIL models; SANP, sentinel application platform; EVI2, enhanced vegetation index 2; SeLI, sentinel-2 LAI index; REIS, RapidEye earth-imaging system; TCARI, transformed chlorophyll absorption in reflectance index; OSAVI, optimized soil-adjusted vegetation index the optimized soil-adjusted vegetation index; TCI, triangular chlorophyll index; and CVI, chlorophyll vegetation index.

### Tuber yield prediction

Achieving the maximum crop yield at the lowest investment is an ultimate goal for farmers (Al-Gaadi et al., 2016). Early detection of problems associated with crop yield can greatly help with the decision-making for field management and reduce profit loss. In addition, yield mapping pre- or post-harvest can be used as spatial references for implementing variable rate technologies to achieve precise applications of field-level inputs and thus optimize production across entire fields (Al-Gaadi et al., 2016).

Potato tuber yield is jointly affected by genetic variation, environmental conditions (soil and weather), seed quality, and crop management practices (Li et al., 2021a). Classical potato yield prediction models are often used to estimate yields within the growing season by considering nitrogen fertilizer, temperature, and daylight or the incidence of solar radiation (Wang X. et al., 2021). Recent studies showed higher prediction accuracies by using machine learning models with remote sensing data at various levels, providing target-specific insights and recommendations for precision agriculture practices.

Salvador et al. (2020) predicted potato tuber yield at a municipal level in Mexica using machine learning models with satellite remote sensing imagery. The models were trained with NDVI values calculated from the TERRA satellite images combined with meteorological data on a monthly basis. The best testing performance was from the Random Forest model for the winter cycle (*R*^2^ = 0.76 and RMSE% = 18.9) and the polynomial Support Vector Machine for the summer cycle (*R*^2^ = 0.86 and RMSE% = 14.9). There are a few similar studies using satellite imagery and machine learning models for predicting potato yield at large scales in Spain (Gómez et al., 2019), Saudi Arabia (Al-Gaadi et al., 2016), and Bangladesh (Bala and Islam, 2009).

Unmanned aerial vehicle-based imagery is largely used for predicting potato yield in site-specific studies. Multi- (Tanabe et al., 2019; Li et al., 2021a) and hyper-spectral (Li B. et al., 2020; Sun et al., 2020) have been used to establish and validate a variety of machine learning models for predicting potato yield. The prediction performance of images collected at different growth stages was compared. It was found that images at earlier growth stages, i.e., the tuber initiation stage (Li et al., 2021a) and vegetative growth stage (Tanabe et al., 2019), tuber bulking stage (Li B. et al., 2020) had better prediction performances than those at the later stage when close to the end of seasons. Even though, Sun et al. (2020) reported a decent prediction accuracy with *R*^2^ = 0.63, RMSE = 3.03 ton/ha, and RMSE% = 5.8 when using hyperspectral image features at tuber maturation stage and machine learning models to predict potato tuber yield under four irrigation levels.

### Potato above-ground biomass estimation

Above-ground biomass is a basic agronomic trait in field investigations and is often used to indicate crop growth status, the effectiveness of agricultural management measures, and the carbon sequestration ability of crops (Li B. et al., 2020). Particularly for potatoes, AGB is one of the most important indicators for calculating the nitrogen nutrition index and diagnosing the potato nitrogen status for precise nitrogen fertilizer management (Jin et al., 2021; Yang et al., 2021). Manual measurement of AGB requires cutting culms at the ground level for a defined portion of the experimental plot, and then weighing before (AGB fresh weight) and after drying (AGB dry weight) in an oven until constant weight (Pask et al., 2012), which is laborious and subject to experimental errors (Jimenez-Berni et al., 2018).

Estimations of potato AGB using remote sensing methods have been explored in a variety of ways, by integrating spectral-based VIs, plant height (manual- or sensor-based) with machine learning, classical and empirical models. Ground-based data acquired on potato canopies by spectroradiometers (Pei et al., 2019; Elsayed et al., 2021b; Yang et al., 2021), RGB, and thermal cameras (Elsayed et al., 2021a) were used as predictors in machine learning models and the studies showed that the agreements between the estimated and measured biomass were up to *R*^2^ of 0.7–0.8. Pei et al. (2019) also compared the estimation performance using the ground spectral data at different stages and the best result was given by the spectral features at the tube growth stage with *R*^2^ = 0.71 and RMSE% = 19.3.

In addition to the spectral features, plant height was considered as an important contributor to potato biomass. ten Harkel et al. (2020) derived the potato plant height from a UAV-LiDAR device and imported it to an empirical model originated from (Jimenez-Berni et al., 2018) for the estimation of biomass from LiDAR. The biomass solely based on plant height had an *R*^2^ of 0.24 with the RMSE of 22.1 g/m^2^. In another case, the UAV image-based plant height (from RGB images using the Structure from Motion method) was integrated with UAV hyperspectral VIs into a Random Forest model. The model accuracy for the biomass fresh weight was up to *R*^2^ = 0.90 with RMSE% = 20.6, and for the biomass dry weight up to *R*^2^ = 0.92 with RMSE% = 17.4 (Li B. et al., 2020).

An interesting comparison in the estimation performance of potato biomass using ground, UAV, and satellite data was conducted by Peng et al. (2021). Data from each source were independently used as variables in a classical model (the Carnegie–Ames–Stanford approach). The results showed that the ground spectral data reached the highest accuracy with *R*^2^ = 0.76 and RMSE% = 22.2, while the UAV and the Satellite multispectral image features had the similar performance with *R*^2^ = 0.70 (RMSE% = 22.4) and *R*^2^ = 0.72 (RMSE% = 24.4), respectively.

### Potato water deficit stress assessment

Potato plants are sensitive to water stress due to their shallow root systems. Approximately 70% of the total water needed for potato growth comes from the upper 30 cm of the soil (Ahmadi et al., 2011). Potato commonly grows in coarse-textured soil with low water holding capacity (Rud et al., 2014) or in arid and semiarid countries (Elsayed et al., 2021a). Therefore, inappropriate water management practices or water deficit stress in potato plants could cause significant reductions in tuber yield (Karam et al., 2014) and degraded tuber quality (Djaman et al., 2021).

The plant water deficit is conventionally detected by measuring plant water statuses, such as leaf water potential and stomatal conductance, or by measuring soil gravimetric water content. All of the measurements are direct, destructive, labor-intensive, and unable for large-scale cultivation or tracking intra-field variability (Rud et al., 2014). CWSI, accounting for the differences between canopy and air temperatures at a fixed air vapor pressure deficit, has been extensively validated to represent plant water status, after it was designed by Idso et al. (1981). The use of leaf or canopy temperature to detect the water-related stresses is based on the principle that a plant’s stomatal closure takes place during water stress, which results in a decrease of energy dissipation and an increase in plant temperature (Idso et al., 1981; Elsayed et al., 2021a). The CWSI has been proposed as a good indicator of plant water stress (Cucho-Padin et al., 2020) and proved relating to stomatal conductance (Rud et al., 2014) and yield (Ghazouani et al., 2017).

The computation of CWSI requires the knowledge of plant canopy temperature which can be acquired through remote sensing technologies. Handheld infrared cameras have been used to obtain temperature values of plant canopy and soil to calculate the CSWI values for potato plants under irrigation treatments. Cucho-Padin et al. (2020) found that the CWSI values derived from a TIR camera had a high correlation (the Pearson’s correlation coefficient *r* = 0.84) with those from ground proximal sensors. The results also showed that no significant reduction in tuber yield was observed by using CWSI < 0.4 as a threshold for irrigation in potato crops, compared to the irrigation amount with CWSI > 0.4, which agrees with other works (Ramírez et al., 2016; Rinza et al., 2019) and provides a reference for saving irrigation water in arid and semiarid regions (Cucho-Padin et al., 2020).

The effectiveness of aerial TIR images in representing potato water status was evaluated in Rud et al. (2014) by comparing the image-derived CWSI with the biophysical measurements of leaf stomatal conductance values. In this study, RGB images were acquired simultaneously with the TIR images and used to create masks for the TIR images to distinguish between vegetation and soil. The CWSI was calculated based on the difference between leaf and air temperatures normalized to the variation in environmental meteorological conditions and showed high correlations (0.64 ≤ *R*^2^ ≤ 0.99) with the stomatal conductance measurements.

A few studies have tried to combine spectral indices with the CWSI and canopy temperature to indicate potato water stress. Elsayed et al. (2021a) reported a good alignment (*R*^2^ = 0.89) between the estimated and measured water content of the potato AGB by using the spectral indices derived from RGB and thermal images combined with machine learning models. Similarly, Gerhards et al. (2016) compared the performance of detecting water content in potato plants between spectral and thermal-based indices. The results showed that the CWSI was most suitable for water stress detection among all the thermal-based indices. Additionally, it was found that the NIR/SWIR reflectance-based indices and the spectral emissivity (non-imaging devices) were equally sensitive to plant water content and responded quicker than the indices from imaging devices (such as the NDVI from a hyperspectral TIR camera; Gerhards et al., 2016).

### Potato nitrogen concentration estimation

Potato growth depends largely on the nitrogen availability in the soil (Nigon et al., 2014). However, the shallow-root system coupled with their common cultivation in coarse-textured soils leads to poor nitrogen use efficiency in potato plants (Nigon et al., 2014). Excessive applications of nitrogen fertilizers, on one hand, might have negative impacts on potato productivity by inducing a greater growth of aboveground biomass but reducing the root growth. On the other hand, it raises the health risks associated with nitrate-contaminated groundwater caused by the high rates of nitrate leaching in irrigated potato fields (Zebarth and Rosen, 2007; Lynch et al., 2012). Therefore, precise spatial and temporal nitrogen management is important to optimize production and minimize environmental nitrogen losses (Nigon et al., 2014).

One of the typical guidance on the timing and amount of nitrogen applications on potato is nitrogen nutrition index, defined as the ratio of the actual nitrogen concentration (N_c_) over the minimum crop nitrogen concentration allowing for maximum biomass production (N_cri_). While N_cri_ is an estimated value depending on the potato AGB, N_c_ needs to be determined along with the potato growth and traditionally by wet tests in laboratory. However, N_c_ values from the sampled potato plants might not accurately represent nitrogen status for an entire field and potato nitrogen status might change considerably by the time of analysis (Zhou et al., 2018). Thus, fast and accurate estimation of potato N_c_ values is urgently needed to assist the precision nitrogen management in potato productions.

Canopy spectral reflectance has been used extensively in agriculture to measure the actual nitrogen status of crop growth in order to guide nitrogen fertilization (Raun et al., 2002; Van Evert et al., 2012; Zhou et al., 2016c). The principle that the canopy spectral reflectance can indicate the canopy N_c_ values is based on the fact that the light reflected in certain wavebands is sensitive to leaf chlorophyll concentration, which in turn closely depends on leaf nitrogen concentration (Lawlor et al., 1989; Baret and Fourty, 1997; Hansen and Schjoerring, 2003). Studies have been conducted to compare the performances in estimating the potato N_c_ among canopy spectral reflectance obtained at different scales (handheld/UAV/Satellite) with narrow or wide spectral bandwidths and various ranges in spectral wavelength. The commonly used spectral features and their details have been summarized in Table 3.

### Potato disease detection

Potato production is threatened by several diseases resulting in considerable yield losses, causing decreases in the quality and increases in the price of potatoes (Taylor et al., 2008). In-season early detection is particularly important for disease management decisions to control disease severity and prevent the spread of diseases (Oppenheim and Shani, 2017). Besides, detecting the overall disease grade of potato fields allows growers to make decisions regarding the best way to store, sell, and manage the fields for future generations (Gibson-Poole et al., 2018). Traditional potato disease inspection requires visual identification of the signs of disease. However, the disease plants can be difficult to identify, especially when symptoms are not yet developed or at the early stages. The remote sensing technique provides an opportunity to overcome the limitations, and they have already been widely used to detect diseases of potatoes for many years.

Among all the diseases that occurred during potato growth, Potato Virus Y (*Potyviridae*, PVY) is the most common reason for the rejection of seed lots from certification and one of the most common potato diseases (Griffel et al., 2018). The foliar symptoms of PVY include mosaic, leaf drop, and interveinal necrosis, and this virus can also cause necrotic rings on potato tubers (Gray et al., 2010). However, the recently prevalent PVY strains exhibit transient and milder foliar symptoms, making it less effective the visual inspection of seed lots with high PVY incidence (Frost et al., 2013). Spectral reflections in the NIR and SWIR regions have provided differentiable profiles in the underlying responses to the PVY stress. Couture et al. (2018) assessed the efficacy of hyperspectral information (a handheld device with narrow bands between 350 to 2,500 nm) in detecting the presence of PVY in potato leaves prior to the onset of the visual symptoms and observed a significant difference in the whole spectral profile between the PVY-negative and PVY-positive plants, especially in the NIR and SWIR spectral regions. A partial-least square discriminate analysis model trained with normalized spectral indices derived from the hyperspectral bands correctly identified the positive PVY infection status *in vivo* with a mean cross-validation accuracy of Kappa = 0.73. Similarly, high accuracy of 89.8% was reported by Griffel et al. (2018) in classifying the PVY-infected and non-infected potato plants using the UAV-based spectral information in the NIR and SWIR regions. Another example was given by Polder et al. (2019) that the healthy potato plants were distinguished from PVY infected ones (the classification Precision = 0.78) by a deep learning model directly using the hyperspectral images (400–1,000 nm).

Potato late blight (*Phytophthora infestans*) is another serious disease affecting potato production worldwide (Hwang et al., 2014). The disease rapidly destroys leaves, and it consequently leads to yield losses and tuber quality deterioration. Symptoms of late blight on potato plants mostly appear on the leaves, developing from small, dark green, irregular-shaped water-soaked spots to large, dark brown or black lesions surrounded by a yellow chlorotic halo. Compared to the PVY, late blight affections are more easily detectable from potato canopies using remote sensing techniques. Various sensing combinations have been evaluated with promising potential to estimate the late blight severity, from ground-based spectrometer (Ray et al., 2011; Franceschini et al., 2017a; Gold et al., 2020) to UAV-based RGB (Sugiura et al., 2016), multi-/hyper-spectral (Nebiker et al., 2016; Franceschini et al., 2017b; Duarte-Carvajalino et al., 2018) cameras and to satellite-based MSI (Dutta et al., 2014). Despite using considerably different prediction models, the general idea of quantifying the late blight severity is first to differentiate the infected and the healthy leaves parts and then obtain as the estimations the percentage of the damaged part over the healthy part (Dutta et al., 2014; Sugiura et al., 2016; Duarte-Carvajalino et al., 2018).

Another potato disease that has been detected using remote sensing techniques is early blight (*Alternaria solani*). Atherton et al. (2015) analyzed the spectral profile of potato plants with the early blight and noticed a significant difference in the spectral reflectance between heavily diseased plants and healthy plants. Van De Vijver et al. (2020) developed a ground-based proximal sensing platform-Hypercart for acquiring hyperspectral images in the potato field. The machine learning models highly agreed (*R*^2^ = 0.92) with the visual assessments. Additionally, significant differences between healthy leaf and injected leaf tissues were observed in the green (550 nm), red (680 nm), and NIR (720–750 nm) regions and the NIR was recognized as the most helpful region for detecting the early blight lesions.

### Other traits

#### Potato plant height

Plant height is one of the most important traits for precision agricultural management. Plant height usually had a significantly positive correlation with yield in many types of crops, thus it is often used as an indicator of crop yield potential (Li et al., 2016; Feng et al., 2019). Besides, plant height can be used to estimate crop AGB and crop lodging. Remote sensing of plant height commonly adopts two different techniques, one directly obtaining the depth information from sensors, for example, the LiDAR devices. The other one is to estimate the 3D information from highly overlapped 2D images using photogrammetric methods such as stereo vision or structure from motion.

The effectiveness and accuracy of plant height measurement in remote sensing depend on the joint effects of the device type, measuring distances, algorithms, and plant variations. For estimating the potato plant height, ten Harkel et al. (2020) used a UAV-LiDAR system at 40 m above ground level and obtained the estimation accuracy of *R*^2^ = 0.50 and RMSE = 12 cm. By using photogrammetric methods, a less expensive way than the LiDAR devices is to take the difference between DSM (representing the absolute elevation of the object in image pixels) and DEMs (representing the absolute elevation of the bare ground under the canopy) derived from RGB imagery. Estimated potato heights often showed high correlations with the *R*^2^ = 0.93 and RMSE = 6.39 cm using a high-resolution RGB camera at a flight height of 30 m (Li B. et al., 2020) and *R*^2^ = 0.52 at a flight height of 75 m with more than 200 potato varieties (De Jesus Colwell et al., 2021).

#### Leaf area index

Leaf area index is one of the most fundamental vegetation biophysical parameters, defined as a dimensionless measure of the one-sided leaf area (m^2^) per unit ground surface area (m^2^). LAI has long been reported as a good indicator of vegetation status (Reyes-González et al., 2019), photosynthesis rate (Clevers et al., 2017), biomass accumulation (Viña et al., 2011), evapotranspiration (Jung et al., 2010), yield estimation (Luo et al., 2020), etc. In potato growth, LAI is also crucial in estimating plant nitrogen concentration. Zhou et al. (2017) developed a ratio vegetation index/LAI-reference curve defining the optimal nitrogen-nutrition for potatoes throughout the season and it could be used as a diagnostic tool to detect nitrogen stress in potato plants during the season.

A variety of handheld instruments have been developed specifically for measuring plant LAI in field conditions, such as the LAI-2000/LAI-2200C instrument (Li-Cor, Inc, Lincoln, NE, United States) the SS1 Sun-Scan canopy analyzer (Delta-T Devices Ltd, Cambridge, United Kingdom), and AccuPAR LP-80 ceptometer (Decagon Devices, Inc). Multi-/hyper-spectral spectrometers are also commonly used to estimate LAI through empirical models that were derived from historical data between LAI measurements (by the LAI instruments) and spectral indices (Herrmann et al., 2011; Papadavid et al., 2011; Clevers et al., 2017). Satellite and UAV-based LAI estimates were validated for measuring at larger scales by observing significant linear relationships with the measurements of the handheld LAI instruments (Herrmann et al., 2011; Clevers et al., 2017; Roosjen et al., 2018).

#### Leaf chlorophyll content

Leaf chlorophyll content (LCC) plays a significant role in plant growth, helping crops to absorb and store the energy to build their tissues. Combining carbon dioxide (absorbed from the air) and water, the stored energy can be converted into glucose through the photosynthesis process (Zheng et al., 2018). Then, the plants utilize the glucose and the nutrients (such as fertilizers and water) from the soil to generate new leaves and other parts, and release the produced oxygen back into the air (Yadav et al., 2010). The LCC is one essential indicator of plant health, and can be used to assess the photosynthetic rate, plant productivity, and nutrient level (Clevers and Kooistra, 2012; Vesali et al., 2017).

A portable non-imaging device, the Soil Plant Analysis Development chlorophyll meter, provides a rapid, accurate, and non-destructive approach to measure the LCC for different crops. Readings from this device have been successfully used as an indicator of plant health status and nitrogen concentrations in potato remote sensing (Giletto et al., 2010; Ramírez et al., 2014, 2015) and often used as ground truth for investigating the effectiveness of RGB and spectral images (in lab conditions) in estimating the LCC, providing the basis for monitoring the LCC *in situ* with the remote sensing platforms for potato management (Dutta Gupta et al., 2013; Borhan et al., 2017). Under field conditions, handheld hyperspectral spectrometer (Liu et al., 2020), satellite multispectral imagery (Kooistra and Clevers, 2016), and UAV hyperspectral imagery (Gevaert et al., 2015) have been explored with promising potential in estimating the potato LLC.

#### Potato emergence detection and plant counting

Potato emergence rate and uniformity play important roles in potato field management (Moran et al., 1997) and yield prediction (Ciuberkis et al., 2007). Poor potato emergence and canopy uniformity lead to variations in the amount of canopy sunlight interception and hence varied photosynthetic efficiency (Moran et al., 1997). Thus, consistent potato emergence is always desirable at early growth stages and is a key trait being routinely assessed in potato agronomy and potato improvement research. Potato emergence can be affected by many factors, such as seed quality, dormancy period, soil temperature, water stress, and nutrient deficiency (Sankaran et al., 2017; Li et al., 2019). The standard method for assessing crop emergence is to visually count the number of plants that emerged at frequent intervals during the establishment phase of development, whereas the uniformity is estimated by subjective and inaccurate manual scoring (Knowles and Knowles, 2006; Herman et al., 2016). Remote sensing techniques can greatly enhance the efficiency in the emergence evaluation. There have been a number of studies leveraging UAV-based imagery and advanced machine learning methods for detecting potato emergence.

Sankaran et al. (2017) observed a significant correlation (*r* = 0.82) between the image-based and manual plant count. Image pixels representing plant canopies were separated (by a color threshold) from the UAV-based multispectral images collected at 15 m in a potato field and then the number of image polygons were estimated as the plant count. Similarly, Li et al. (2019) obtained the image polygons from the UAV-based RGB images using the Otsu thresholding method. The potato emergence rate of a pixel polygon was then estimated from six morphological features of the polygon by a trained machine learning model. The estimations showed a high correlation (*R*^2^ = 0.96) with the manual count. Despite the excellent performance, the image plant counts in these two studies required the accurate separation of pixels between plant and background, which limits its applications for situations with non-uniform backgrounds and changing illuminations. A more adaptable way was proposed by Machefer et al. (2020) for potato plant counting by directly inputting UAV-based RGB images to a transferred deep learning architecture. The model was previously trained on low-density crops so a much smaller number of labeled images was needed to train the model for potato plants. Besides, no pre-processing step was required on the UAV images, which helps to keep the stable performance under varying UAV image collection conditions.

## Conclusion

Remote sensing has great potential in characterizing potato traits for precision agricultural applications, as a promising alternative to conventional field scouting and visual examination for farmers. This review fills a critical gap in precision agriculture literature by providing a comprehensive review of the applications of remote sensing technologies specifically used in potato trait characterization. We reviewed the commonly used imaging sensors and remote sensing platforms with the comparisons of their strengths and limitations in characterizing potato traits. The main applications that are benefited from the development of advanced remote sensing technologies have been summarized with the scientific logic behind the scenes and the current research status. The applications include tuber yield prediction, AGB estimation, water stress detection, nitrogen stress assessment, and disease detection. Four other agronomic traits that were widely investigated relating to potato growth were listed as well, namely plant height, LAI, LCC, and emergence rate and plant counting. This review will update potato agronomists and farmers with the latest approaches and research outcomes, as well as provide a selective list for those who have the intentions to apply remote sensing technologies to characterize potato traits for precision agriculture.

## Author contributions

CS: conceptualization and writing—original draft preparation. JZ, YM, YX, and BP: writing—reviewing and editing. ZZ: conceptualization, resources, and writing—reviewing and editing. All authors contributed to the article and approved the submitted version.

## Conflict of Interest

The authors declare that the research was conducted in the absence of any commercial or financial relationships that could be construed as a potential conflict of interest.

## Publisher’s Note

All claims expressed in this article are solely those of the authors and do not necessarily represent those of their affiliated organizations, or those of the publisher, the editors and the reviewers. Any product that may be evaluated in this article, or claim that may be made by its manufacturer, is not guaranteed or endorsed by the publisher.
